# The PHQ-PD as a Screening Tool for Panic Disorder in the Primary Care Setting in Spain

**DOI:** 10.1371/journal.pone.0161145

**Published:** 2016-08-15

**Authors:** Roger Muñoz-Navarro, Antonio Cano-Vindel, Cristina Mae Wood, Paloma Ruíz-Rodríguez, Leonardo Adrián Medrano, Joaquín T Limonero, Patricia Tomás-Tomás, Irene Gracia-Gracia, Esperanza Dongil-Collado, M. Iciar Iruarrizaga, PsicAP Research Group

**Affiliations:** 1 PsicAP Research Group, Complutense University of Madrid, Madrid, Spain; 2 Faculty of Psychology, University of Valencia, Valencia, Spain; 3 Faculty of Psychology, Complutense University of Madrid, Madrid, Spain; 4 Castilla La Nueva Primary Care Center, Health Service of Madrid, Madrid, Spain; 5 Faculty of Psychology, University Siglo 21, Córdoba, Argentina, Spain; 6 Stress and Health Research Group, Faculty of Psychology, Autonomous University of Barcelona, Barcelona, Bellaterra, Spain; 7 Malva-Rosa Primary Care Center, Health Service of Valencia, Valencia, Spain; 8 Faculty of Psychology, Catholic University of Valencia, Valencia, Spain; University Of São Paulo, BRAZIL

## Abstract

**Introduction:**

Panic disorder is a common anxiety disorder and is highly prevalent in Spanish primary care centres. The use of validated tools can improve the detection of panic disorder in primary care populations, thus enabling referral for specialized treatment. The aim of this study is to determine the accuracy of the *Patient Health Questionnaire-Panic Disorder* (PHQ-PD) as a screening and diagnostic tool for panic disorder in Spanish primary care centres.

**Method:**

We compared the psychometric properties of the PHQ-PD to the reference standard, the Structured Clinical Interview for DSM-IV Axis I Disorders (SCID-I) interview. General practitioners referred 178 patients who completed the entire PHQ test, including the PHQ-PD, to undergo the SCID-I. The sensitivity, specificity, positive and negative predictive values and positive and negative likelihood ratios of the PHQ-PD were assessed.

**Results:**

The operating characteristics of the PHQ-PD are moderate. The best cut-off score was 5 (sensitivity .77, specificity .72). Modifications to the questionnaire's algorithms improved test characteristics (sensitivity .77, specificity .72) compared to the original algorithm. The screening question alone yielded the highest sensitivity score (.83).

**Conclusion:**

Although the modified algorithm of the PHQ-PD only yielded moderate results as a diagnostic test for panic disorder, it was better than the original. Using only the first question of the PHQ-PD showed the best psychometric properties (sensitivity). Based on these findings, we suggest the use of the screening questions for screening purposes and the modified algorithm for diagnostic purposes.

## Introduction

Panic disorder (PD) has a high comorbidity (70%) with other anxiety disorders and is the most disabling of all [1]. Like many anxiety disorders, PD is also associated with numerous physiological disorders, including digestive problems, high blood pressure, cardiovascular risk, headaches, heart disease, and musculoskeletal disorders [2]. PD is also associated with psychological disorders, including depression, social phobia, and a high suicide rate [3].

According to a meta-analysis of 12 general population studies in Europe, the annual prevalence of PD is 1.8%, ranging from 0.7% to 3.1% [4]. In the European Study of the Epidemiology of Mental Disorders (ESEMeD), the annual prevalence of PD in Europe and Spain, respectively, was 0.8% and 0.6%, with a lifetime prevalence of 2.1% and 1.7% [5]. Another study conducted in Spain reported the same annual prevalence rate (0.6% in the general population) versus 7% in Spanish Primary Care (PC) centres [6]. An older study, which used the PRIME-MD test [7] to assess the prevalence of PD in PC centres in Spain, reported a 2.2% rate.

In previous years, other studies conducted in European countries using tests such as the Patient Health Questionnaire (PHQ) [8] have reported a prevalence rate of 9% in Spain [9], with a prevalence rate in women that was nearly double that of men: 11.6% vs. 5.9%, respectively. Another study conducted in Spain using the PRIME-MD test reported an annual prevalence of 9.7% (11.5% for women, 6.8% for men) [3]. However, when diagnostic interviews were used, prevalence rates were slightly lower, as Serrano-Blanco et al. [6] found. Those author reported an annual prevalence of 8.8% and a current prevalence of 7% (men, 3.9%; women, 8.8%). The higher prevalence in women in Spain is consistent with reports from other countries, including the United States [10].

The PHQ—specifically, the PD subscale (PHQ-PD)—is one of the main diagnostic tools used to assess PD. In the original study [8], the authors evaluated the operating characteristics of the test, finding a sensitivity of .81—substantially higher than the same measure obtained with the PRIME-MD test—and a specificity of .99, identical to the PRIME-MD. Subjects who responded affirmatively to the first 4 questions on the test and report 4 or more symptoms are considered to have a probable diagnosis of PD. In the Spanish validation study, the sensitivity was .83 and the specificity was .98, similar to the values obtained on the original test [7]. Despite the good sensitivity and specificity of this test for PD, the currently available evidence to support the psychometric properties of the PHQ-PD is inconclusive. As a result, many authors have modified the algorithm to obtain better psychometric properties [11–13]. Löwe et al. [11] assessed a sample of 499 patients using the original PHQ algorithm (affirmative response on the first 4 questions and 4 or more somatic symptoms), finding a sensitivity of .75 and a specificity of .96. When they modified the algorithm to 3 affirmative responses on the first 4 questions and 4 or more somatic symptoms, they found that sensitivity improved to .86 while specificity decreased slightly, to .91. Finally, when they changed the algorithm to match those of the SCID-I criteria of the DSM-IV Axis I (2 affirmative answers on the first 4 questions ≥ 4 or more somatic symptoms), they found a sensitivity of .91 and a specificity of .88. Wittkampf et al. [13] used the PHQ-PD to assess a total sample of 479 PC patients at high risk for mental health problems, finding a sensitivity and specificity of .44 and .94, respectively, with the original PHQ criteria. When they changed the criteria to 3 affirmative answers on the first 4 questions and 4 or more somatic symptoms, the sensitivity increased to .61 while the specificity fell slightly (to .89). Finally, using the criteria of 2 affirmative answers on the first 4 questions ≥ 4 or more somatic symptoms, they found a sensitivity of .66 and a specificity of .87.

The original PHQ criteria are the strictest and therefore have the lowest sensitivity scores. This is problematic given that the role of screening tests in primary care is to identify potential patients who may merit further study. In the primary care setting, screening tools need to be highly sensitive to reduce the level of false-negatives, even though this implies a higher rate of false-positives. In this context, the aim of the present study was to determine the optimal PHQ-PD psychometric properties in a primary care patient sample in Spain. We evaluated the psychometric properties of the PHQ-PD as a screening and diagnostic tool for PD in users of PC services and compared the results to the Structured Clinical Interview for DSM-IV Axis I Disorders (SCID-I)—a diagnostic interview for DSM-IV Axis I diagnosis [14]—which was used as the reference standard.

## Method

### Study population

#### Setting

The study was conducted between January and December 2014 in a sample of PC patients aged 18–65 years of age (mean age, 44 years ±11.87) of the large PsicAP Project, a randomized clinical trial conducted in 22 PC centres of the public health system in Spain (ISRCTN58437086). We selected five centres from among these 22 centres located in 17 different autonomous communities in Spain (two centres in Valencia, and one each in Albacete, Biscay and Mallorca) from which the sample was recruited.

#### Patients

In Spain, all users of the public health system are assigned a General Practitioner (GP) at their local primary care centre. PC is the gateway to the healthcare system for all patients and acts as a bridge between other specialized services. All patients (*n* = 298) who presented at one of these five PC centres during the study period with somatic or psychological complaints, such as feelings of sadness, worries, sleeping problems or elevated distress, were invited to participate in the study by their GPs. Of this initial sample, 38 subjects were excluded, as follows: 20 patients (6.7%) were not reachable, 9 (3%) did not meet the age range criteria, 6 (2%) dropped out voluntarily, and 3 (1%) were excluded for other reasons. Finally, 260 participants voluntarily agreed to participate and signed the informed consent form after being fully informed about the study. All 260 subjects completed the PHQ and 178 also completed the SCID-I. The participants who completed both the PHQ and the SCID-I were considered comparable (*p* >.35) in terms of socio-demographic variables for the whole sample. There was only a slight increase in the relative dropout rate in the Biscay centre versus the other centres. See Table 1 for more details.

**Table 1:** Demographics and medication of total and SCID-I completed samples.

	Total simple (*n* = 260)		SCID-I completed (*n* = 178)	
	*n*	%	*n*	%
Primary Care Center				
Albacete	39	15.0	21	11.8
Mallorca	33	12.7	30	16.9
Valencia	155	59.6	122	68.5
Biscay	33	12.7	5	2.8
Sex				
Female	186	71.5	125	70.2
Male	74	28.5	53	29.8
Marital status				
Married	130	50.0	86	48.3
Divorced	28	10.8	21	11.8
Widowed	5	1.9	3	1.7
Separated	19	7.3	14	7.9
Never married	48	18.5	29	16.3
Unmarried	30	11.5	25	14.0
Level of education				
No schooling	7	2.7	4	2.2
Basic education	94	36.2	71	39.9
Secondary education	40	15.4	27	15.2
High School	64	24.6	46	25.8
Bachelor	47	18.1	27	15.2
Master/doctorate	8	3.1	3	1.7
Employment situation				
Part-time employee	28	10.8	18	10.1
Employed full time	85	32.7	58	32.6
Unemployed, in search of work	77	29.6	52	29.2
Unemployed, not looking for work	36	13.8	27	15.2
Temporary low labor	14	5.4	11	6.2
Permanent low labor	4	1.5	2	1.1
Retired	16	6.2	10	5.6
Income level				
Less than 12,000	119	45.8	87	48.9
12,000 to 24,000	112	43.1	79	44.4
Between 24,000 and 36,000	20	7.7	10	5.6
More than 36,000	9	3.5	2	1.1
Hypnotics				
No	147	56.5	100	56.2
Yes	113	43.5	78	43.8
Anxiolytics/tranquilizers				
No	175	67.3	119	66.9
Yes	85	32.7	59	33.1
Anti-depressants				
No	194	74.6	126	70.8
Yes	66	25.4	52	29.2

The inclusion criteria were: male and female adults between 18 and 65 years, inclusive, who sought treatment for anxiety, depression and/or somatic symptom disorder during the study inclusion period at any of the five PC centres. Exclusion criteria were the presence of severe mood disorders (e.g., bipolar disorder, or a severe major depressive disorder), substance abuse or dependence, any other severe mental disorder (e.g., personality disorder, mental retardation), a history of frequent or recent suicide attempt(s), a high level of disability, not proficient in Spanish, or participation in another clinical trial.

### Measures

We collected the following demographic variables: age; sex; marital status; level of education; employment situation; income level; and medical history. In addition, participants completed the following scales:

#### Patient Health Questionnaire (PHQ)

The PHQ [8] is a self-report screening test derived from the PRIME-MD test [15], a two-stage evaluation system of mental disorders in PC containing the Patient Questionnaire and the Physicians’ Clinical Evaluation Guide. The PHQ includes sections examining somatization (PHQ-15), depressive disorder (PHQ-9), PD (PHQ-PD), generalized anxiety disorder (GAD-7), eating disorders and alcohol-related disorder.

#### Patient Health Questionnaire-Panic Disorder (PHQ-PD)

The PD section is a part of PHQ [8] which comprises 15 items (questions 3a-d and 4a-d-k.) Question 3 includes elements of the DSM-IV classification system to review the history and frequency of panic attacks (item 3a: “In the last 4 weeks, did you have an anxiety attack—sudden feeling of fear or panic?”). Question 4 contains information related to somatic symptoms of panic attacks (item 4a: “shortness of breath” or item 4i: “tingling or numbness in parts of the body”). There are two answer categories: "no" (0 points) and "yes" (1 point.)

Patients are considered to have a positive score on the PD section if all four parts of question 3 (a-d) are answered affirmatively (4 points) together with ≥ four items for question 4 about somatic symptoms (4 additional points). However, since the primary goal of screening tools is to achieve a high detection rate, we made several modifications of this assessment algorithm in an attempt to increase the test's sensitivity to detect PD. The original assessment algorithm requires that the first four answers to question 3 be positive. By contrast, our modified algorithm required a positive answer on the first item (3a) and one positive answer on items 3b, 3c, or 3d.

The only screening question we tested for its diagnostic validity was question 3a from the PHQ-PD: "In the last 4 weeks, did you have an anxiety attack—sudden feeling of fear or panic?”

#### Structured Clinical Interview for DSM-IV Axis I Disorders

The SCID-I [14] consists of a semi-structured interview to diagnose mental disorders based on Axis-I DSM-IV criteria. In our study, all researchers were trained in the use of SCID-I by an expert clinical psychologist prior to administering the test to patients. During the study, all interviewers had training sessions under the supervision of the expert. Patients who met the following criteria were diagnosed with PD: a) fulfil all nine DSM-IV criteria during the previous two weeks; b) present positive scores for at least one of the first two symptoms of PD and present ≥ 5 of all the symptoms.

### Procedure

Patients with anxious, depressive or physical symptoms without a clear biological basis were asked by the GP to participate. They were given the Patient Information Sheet, with detailed information about the study, and asked to sign the Informed Consent Form. Once the form was signed, a meeting was arranged to review the study details and to complete the PHQ and other tests. Computerized versions of the tests were used in most cases; however, patients with impaired vision received assistance with the questionnaires as needed. Paper versions of the tests were provided to patients who had difficulties using the computer. Next, participants were scheduled for the SCID-I. Prior to application of the SCID-I, all participants received a Patient Information Sheet and signed an informed consent form. A trained psychologist blinded to the results of the PHQ-PD conducted the interviews. We use the SCID-I module of PD as the gold standard to confirm the diagnosis of PD in our sample. Then we compared the results of the SCID-I with those obtained with the PHQ-PD.

### Ethical aspects

The study was conducted in accordance with Declaration of Helsinki. This project has been promoted by the *Psicofundación* (Spanish Foundation for the Promotion, Scientific and Professional Development of Psychology) and approved by the Corporate Clinical Research Ethics Committee of Primary Care of Valencia (CEIC-APCV), Spain, as the national research ethics committee coordinator, and the Spanish Medicines and Health Products Agency (AEMPS). The study was also approved by the five participating PC centres: The CEIC-APCV, the Clinical Research Ethics Committee of the Hospital Universitario de Albacete (CEIC-HUA), the Clinical Research Ethics Committee of Euskadi (CEIC-E), and the Clinical Ethics Committee of the Balearic Islands (CEI-IB).

The study was conducted in accordance with the Spanish Data Security Law. All professionals participating in the study agreed to adhere to the Helsinki Declaration and to Spanish law. Patient participation in the study was completely voluntary and participants were able to withdraw at any time without explanation and without negative consequences for future medical care. No data was made publicly available.

### Statistical analysis

To provide criterion validity of the PHQ-PD, receiver operating characteristic (ROC) curve analysis (sensitivity, specificity, positive and negative predictive values, and positive and negative likelihood ratios) were calculated using different scoring criteria, including the original algorithmic criteria. To evaluate the test's screening properties, we used the sum scores of the PHQ-PD; to assess the diagnostic properties, we used the diagnostic cut-off value. Additionally, differences in the sensitivities and specificities of the original algorithm compared to the two modified algorithms and to the screening question were tested using McNemar's Χ2 tests with the Bonferonni—Holm procedure to adjust for multiplicity. The optimal cut-off value to balance sensitivity and specificity was identified as the value corresponding to the maximum value of Youden’s index, calculated as (sensitivity + specificity– 1).

## Results

### Diagnosis using PHQ

Among the 260 patients that completed the PHQ, more than half were diagnosed with somatization disorders (SD) (PHQ-15 ≥5). Based on the DSM-IV diagnostic algorithm, a large proportion were diagnosed with major depressive disorder (MDD). Similarly, a large proportion of subjects presented MDD according to the PHQ-9 (scores ≥10). In addition, a high percentage presented generalized anxiety disorder (GAD) (GAD-7 ≥10). A smaller percentage of patients diagnosed with PD according to the PHQ-PD algorithm also presented comorbid eating disorder and alcohol-related disorder. No significant differences in socio-demographic variables were observed among the overall patient sample, nor among the 178 who participated in the clinical interview. See Table 2 (upper section) for details.

**Table 2:** PHQ diagnoses and comorbidity of total and SCID-I completed samples.

	Total simple (*n* = 260)		SCID-I completed (*n* = 178)	
	*n*	%	*n*	%
Somatoform disorder (SD)				
Without SD	119	45.8	84	47.2
With SD	141	54.2	94	52.8
Major depressive disorder (MDD)				
Without MDD (Algorithm)	82	31.5	54	30.3
With MDD (Algorithm)	178	68.5	124	69.7
Without MDD (≤ 10)	57	21.9	40	22.5
With MDD (≤ 10)	203	78.1	138	77.5
Panic disorder (PD)				
Without PD (Algorithm 4+4)	203	78.1	138	77.5
With PD (Algorithm 4+4)	57	21.9	40	22.5
Without PD (Algorithm 1+1+4)	150	57.7	104	58.4
With PD (Algorithm 1+1+4)	110	42.3	74	41.6
General anxiety disorder (GAD)				
Without GAD (≤ 10)	80	30.8	50	28.1
With GAD (≤ 10)	180	69.2	128	71.9
Eating disorder				
Without eating disorder	215	82.7	148	83.1
With eating disorder	45	17.3	30	16.9
Alcohol abuse				
Without alcohol abuse	222	85.4	153	86.0
With alcohol abuse	38	14.6	25	14.0
Comorbidity				
MDD + GAD	150	57.7	107	60.1
MDD + SD	115	44.2	81	45.5
GAD + SD	117	45.0	81	45.5
MDD + GAD + SD	104	40.0	74	41.6
GAD + PD	45	17.3	33	18.5
MDD + PD	40	15.4	30	16.9
MDD + GAD + PD	37	14.2	29	16.3
PD + SD	42	16.2	27	15.2
SD + GAD + PD	36	13.8	25	14.0
MDD + SD + PD	34	13.1	23	12.9
SD + MDD + PD + GAD	32	12.3	22	12.4
SD + MDD + PD + GAD + Eating + Alcohol	1	0.4	1	0.3

Note. SD = somatoform disorder, MDD = major depressive disorder, PD = panic disorder, GAD = general anxiety disorder, Eating = eating disorder, Alcohol = alcohol abuse. Comorbidity categories are not exclusive (e.g., “MDD + GAD” comprises “MDD + GAD + SD”)

As expected, we found high comorbidity between disorders. The prevalence of comorbid MDD and GAD was particularly high (150 patients: 57.7%), as was comorbid MDD and SD (115 patients; 44.2%) and comorbid GAD and SD (117 patients; 45%). In addition, 40% of patients had comorbid MDD, GAD and SD. The percentage of patients with comorbid PD and GAD and comorbid MDD and SD was appreciably lower. See Table 2 (lower section) for more details.

### Operating characteristics of PHQ-PD as a screening test

**Fig 1:**
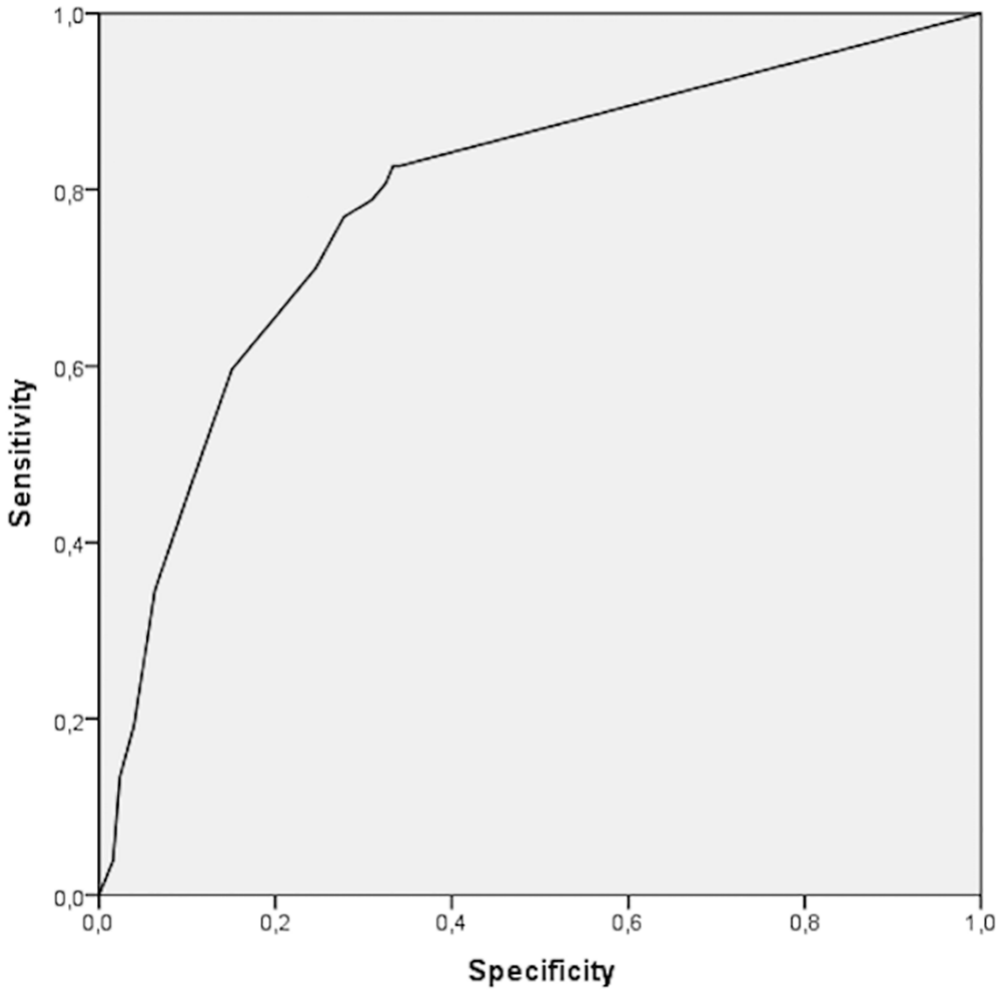
ROC curves for determining the sensitivity and specificity of the overall assessment of the PHD-PD scale.

To establish the clinical utility of PHQ-PD, we drew ROC curves to determine the optimal cut-off score to establish a diagnosis of PD. The ROC curves are valuable because they permit visual inspection of the balance between the sensitivity and specificity of a test; moreover, these curves indicate the level at which the model distinguishes between individuals in whom PD is present and those in whom it is not. Thus, the cut-off level in the curve was the level that provided the optimal relationship between sensitivity and specificity. ROC curve analyses showed that the PHQ-PD performed well, with an area under the curve (AUC) of .79 (Fig 1). The visual analysis scale allows us to analyse various cut-off values corresponding to different specificity and sensitivity values. As shown in Youden’s index, the most appropriate cut-off for PD was 5 (J = .49), with a sensitivity of .77, a specificity of .72, a positive predictive value of .53, a negative predictive value of .88, and positive and negative likelihood ratios of 2.77 and .32, respectively. Most patients (77%) with a SCID-I diagnosis of PD had a score of 5 or more, while most patients (72%) without a SCID-I PD diagnosis scored below this cut-off value. Table 3 shows the various cut-off values.

**Table 3:** Operational characteristics of the PHQ-TP, sensitivity and specificity, positive and negative predictive value, and positive and negative likelihood ratio.

Cut-off Score ROC	Sensitivity	McNemar X2 p-value*	Specificity	Positive Predictive Value	Negative Predictive Value	Positive Likelihood Ratio	Negative Likelihood Ratio	Youden´s Index (J)
PHQ-PD ≥ 4	.79 (.66−.88)		.69 (.61−.76)	.51	.89	2.55 (1.89−3.43)	.31 (.18−.52)	.48
PHQ-PD ≥ 5	.77 (.64−.86)		.72 (.64−.79)	.53	.88	2.77 (2.01−3.81)	.32 (.19−.53)	.49
PHQ-PD ≥ 6	.71 (.58−.82)		.75 (.67−.82)	.54	.86	2.89 (2.04−4.11)	.38 (.25−.59)	.46
PHQ-PD ≥ 7	.65 (.52−.77)		.80 (.72−.86)	.58	.85	3.30 (2.20−4.93)	.43 (.29−.63)	.45
PHQ-PD ≥ 8	.60 (.46−.72)		.85 (.77−.90)	.62	.84	3.95 (2.47−6.33)	.48 (.34−.67)	.45
Original Algorithm ^a^	.50 (.37−.63)	-	.89 (.82−.93)	.65	.81	4.50 (2.56−7.91)	.56 (.43−.74)	.39
Modified Algorithm ^b^	.77 (.64−.86)	.001	.72 (.64−.79)	.53	.88	2.77 (2.01−3.81)	.32 (.19−.53)	.49
Screening Question ^c^	.83 (.70−.91)	.001	.66 (.57−.74)	.50	.90	2.42 (1.85−3.18)	.26 (.14−.48)	.49

* p-values are corrected for multiplicity using the Bonferonni—Holm procedure.

^a^ All of the first four questions are answered with “yes,” and presence of four or more somatic symptoms during an anxiety attack

^b^ At least two of the first four questions are answered with “yes,” other coding criteria unchanged.

^c^ "In the last four weeks, have you had an anxiety attack—suddenly feeling fear or panic?”.

When we analysed the characteristics of the first PHQ-PD screening question (*In the last two weeks*, *did you have an anxiety attack or sudden feeling of panic*?*)*, we found an AUC of .74, a reasonable result. The sensitivity significantly increased (p < .001) against the original and even the modified algorithm, but the specificity decreased. The sensitivity was .83 and specificity .66, a positive predictive value of .50, a negative predictive value of .88, a positive likelihood ratio of 2.42 and a negative likelihood ratio of .32. (See Table 3 for other possible cut-off points and confidence intervals). Youden’s index showed a good performance (J = .49)

### Operating characteristics of PHQ-PD as a diagnostic test

As shown in Table 3, the original algorithm showed an AUC of .69 (a poor result), whereas the modified algorithm showed an AUC of .74, a much better performance. The sensitivity and specificity achieved with the original PHQ-PD algorithm were .50 and .89, respectively. By contrast, with the modified algorithm, the sensitivity improved to .77, while the specificity decreased to .72. With the original algorithm, the positive and predictive values were, respectively, .65 and .81, and the positive and negative likelihood ratios were 4.50 and .56, respectively. With the modified algorithm, the positive and negative predictive value were .53 and .88, respectively, and the positive and negative likelihood ratios were, respectively, 2.77 and .32 (see Table 3 for details, including confidence intervals and alternative cut-off points). Also, Youden’s index showed that the modified algorithm was better (J = .49) than the original algorithm (J = .39).

## Discussion

Emotional disorders such panic or anxiety disorders are common in the community, and are typically highly comorbid on presentation, and often affect functioning [3.] In this study, we tested the psychometric properties of the PHQ-PD, a screening tool to detect panic disorder in Spanish primary care centres. We found a moderate performance for the test when we modified the original diagnostic algorithm, a finding that may help the GP to more easily screen for this emotional disorder.

In Spain, patients with emotional disorders like PD are assessed by their GP who, after only a brief consultation, must decide whether specialized care is required or not [16]. To facilitate this decision-making process for the GP, adequate screening tools with good psychometric properties are needed to identify patients who are candidates for referral (positive screening test result) to a specialized service (psychological or psychiatric treatment).

We found that results of the PHQ-PD correlated well with the PD section of the SCID-I, leading us to conclude that the PHQ-PD is a valid, useful screening tool for this disorder in our sample. The modified algorithm significantly improved the tool's sensitivity versus the original PHQ algorithm (.75 vs .42), albeit with a decreased specificity (.72 vs .86). As mentioned previously, high sensitivity is essential in a screening test, and several authors have found that the sensitivity of the PHQ-PD can be increased by modifying the algorithm [11,13]. Löwe et al. [11] evaluated the properties of the PHQ-PD in a sample of German patients, finding a sensitivity of .75 and specificity of .96; when those authors modified the algorithm in exactly the same way that we did (i.e., positive answer on the first item and a positive answer in any of the three subsequent items, plus 4 or more somatic symptoms), the sensitivity improved to .91 (specificity, .88). Wittkampf et al. [13] reported results that were very similar to ours using the Dutch version of the PHQ-PD Dutch. For the original algorithm, they found a sensitivity of .44 and a specificity of .94 with positive and negative predictive values, respectively, of .30 and .97. These results were similar to those reported by Becker, Zaid & Faris [17], who used the SCID-I as a reference test in a sample of 173 PC patients; in that study, the sensitivity was .47 and the specificity, .96. Although we obtained a similar sensitivity (.42), specificity was lower in our study (.86). Wittkampf et al. [13] modified the algorithm to match the SCID-I criteria, finding that this change increased the test's sensitivity to .66 with a small decrease in specificity (.87). By contrast, when we modified the algorithm to match the SCID-I criteria, we obtained a higher sensitivity (.75) but a lower specificity (.72). Thus, we obtained a higher sensitivity than Wittkampf et al. [13], who reported a sensitivity that was very close to the original algorithm, but this increased sensitivity was achieved at the expense of a lower specificity (which decreased from .86 to .72). Our results are consistent with previous reports in which the PHQ-PD was administered alone (i.e., not in conjunction with the SCID-I) and in which changes in the algorithm improved its sensitivity [11,13,17]. As these authors note, variability among studies in terms of sensitivity may be due to the relatively low prevalence of PD, which leads to large confidence intervals and thus greater variability. In this sense, the Spanish version of the PHQ developed by Díez-Quevedo et al. [18] had a sensitivity of .83 and a specificity of .98—very high scores that may be attributable to the low prevalence of PD.

The SCID-I uses diagnostic criteria that are similar to the modified algorithm used in our study (i.e., an affirmative answer to the first question regarding an anxiety attack or sudden feeling of panic, plus an affirmative answer to one of the three subsequent questions plus ≥ four or more somatic panic symptoms). This same modification to the algorithm significantly improved sensitivity in several studies [11, 13], including the present report. This finding suggests that the PHQ-PD can be used in primary care centres in the public health system to accurately screen for individuals with possible PD. The modified algorithm significantly increases the test's sensitivity, the most important feature of any screening test. However, because this increased sensitivity could cause a major reduction in false-negatives, the specificity of the test is lower and more false-positives are likely.

It is important to emphasize that the screening question (“In the last two weeks, did you have an anxiety attack or sudden feeling of panic?”) showed the best sensitivity but the lowest specificity. Our data suggest that it would be advisable for the GP to administer the full PHQ-PD only in patients who answer the screening question in the affirmative. In addition, due the fact the SCID-I has good specificity, this should be performed after the PHQ-PD test to assure an accurate PD diagnosis. Nevertheless, the use of the PHQ-PD alone should help to improve detection of PD.

### Study limitations

A potential limitation of this study is that the PC centres were not randomly selected. Nevertheless, given that these centres were located in different geographical areas around Spain, we believe that this mitigates the lack of randomization. Moreover, we found no statistically significant differences in terms of results (Table 1) among the various PC centres included in the study, suggesting that lack of randomization had no impact on the study outcomes.

## Conclusions

The findings presented here, when considered together with the outcomes from similar studies, suggest that the PHQ-PD is an adequate screening tool for the detection of PD in Spanish PC centres that share similar characteristic along the Spanish geography.

The PHQ-PD presents many advantages: it has good psychometric properties, and it is short, easy to understand and can be administered without increasing emotional distress in patients. We believe that these data support the use of this tool in primary care centres to help detect PD.
